# Role of amygdala kisspeptin in pubertal timing in female rats

**DOI:** 10.1371/journal.pone.0183596

**Published:** 2017-08-28

**Authors:** Daniel A. Adekunbi, Xiao Feng Li, Shengyun Li, Olufeyi A. Adegoke, Bolanle O. Iranloye, Ayodele O. Morakinyo, Stafford L. Lightman, Paul D. Taylor, Lucilla Poston, Kevin T. O’Byrne

**Affiliations:** 1 Division of Women’s Health, Faculty of Life Sciences and Medicine, King's College London, London, United Kingdom; 2 Department of Physiology, College of Medicine, University of Lagos, Lagos, Nigeria; 3 Henry Wellcome Laboratory for Integrative Neuroscience and Endocrinology, University of Bristol, Bristol, United Kingdom; Universite de Rouen, FRANCE

## Abstract

To investigate the mechanism by which maternal obesity disrupts reproductive function in offspring, we examined *Kiss1* expression in the hypothalamic arcuate (ARC) and anteroventral periventricular (AVPV) nuclei, and posterodorsal medial amygdala (MePD) of pre-pubertal and young adult offspring. Sprague-Dawley rats were fed either a standard or energy-dense diet for six weeks prior to mating and throughout pregnancy and lactation. Male and female offspring were weaned onto normal diet on postnatal day (pnd) 21. Brains were collected on pnd 30 or 100 for qRT-PCR to determine *Kiss1* mRNA levels. Maternal obesity increased *Kiss1* mRNA expression in the MePD of pre-pubertal male and female offspring, whereas *Kiss1* expression was not affected in the ARC or AVPV at this age. Maternal obesity reduced *Kiss1* expression in all three brain regions of 3 month old female offspring, but only in MePD of males. The role of MePD kisspeptin on puberty, estrous cyclicity and preovulatory LH surges was assessed directly in a separate group of post-weanling and young adult female rats exposed to a normal diet throughout their life course. Bilateral intra-MePD cannulae connected to osmotic mini-pumps for delivery of kisspeptin receptor antagonist (Peptide 234 for 14 days) were chronically implanted on pnd 21 or 100. Antagonism of MePD kisspeptin delayed puberty onset, disrupted estrous cyclicity and reduced the incidence of LH surges. These data show that the MePD plays a key role in pubertal timing and ovulation and that maternal obesity may act via amygdala kisspeptin signaling to influence reproductive function in the offspring.

## Introduction

The role of the early life nutritional environment on reproductive development and function has received considerable attention in light of growing concern over the increasing number of women of reproductive age who start pregnancy overweight or obese and/or gain excess weight during pregnancy [1]. Other than adverse influences on pregnancy outcome, maternal obesity has emerged as a risk factor for precocious puberty in girls [2,3] and boys [4] born to obese women and this association between maternal obesity and age at menarche may be independent of pre-pubertal or birth weight. Similarly, studies in rodents have shown that exposure to maternal obesity accelerates sexual maturation in the offspring [5,6], which may have consequences for reproductive function later in life. Impaired growth of ovarian follicles, prolonged or persistent estrus, reduced sperm quality and fertility, lower levels of male reproductive hormones and altered sexual behaviour have been reported in offspring exposed to maternal obesity in early life [7–11].

Despite these observations, little is known of the underlying mechanism by which intrauterine obesogenic environments influence reproductive function in the offspring. There is however substantial evidence for the role of kisspeptin as a potent neuroendocrine regulator of the reproductive system [12,13]. Kisspeptin modulates the secretion of gonadotropin-releasing hormone (GnRH) and mutations in the genes for kisspeptin (*Kiss1*) or kisspeptin receptor (*Kiss1r*) cause impaired puberty progression and infertility [14]. Kisspeptin neurons are in synaptic connection with a subgroup of GnRH neurons as early as embryonic day 16.5 in the mouse [15], raising the possibility of kisspeptin signaling system being operational before birth. Maternal obesity has been associated with elevated endogenous estradiol levels in the offspring at birth, through young adolescent age, and such early life estrogenic exposure may be critical for altered reproductive function later in life [16]. We therefore hypothesized that maternal obesity may modulate central drivers of the reproductive axis, specifically *Kiss1* expression to influence reproductive function in the offspring.

The major focus of studies on kisspeptin signaling has been on the hypothalamus given the abundance of kisspeptin neurons in the arcuate (ARC) and anteroventral periventricular (AVPV) nuclei [17,18]. The expression of *Kiss1* in the medial amygdala particularly in its posterodorsal nucleus [19,20] has also generated interest in extra-hypothalamic roles of kisspeptin. The posterodorsal nucleus of the medial amygdala (MePD) is highly enriched with sex steroid receptors [21,22] and MePD *Kiss1* expression is steroid-dependent, analogous to *Kiss1* expression in the AVPV and ARC [19]. Intra-medial amygdala injection of kisspeptin-10 dose-dependently increases luteinizing hormone (LH) secretion, while kisspeptin receptor antagonism reduces LH pulse frequency [23], thus affirming the influence of this extra-hypothalamic kisspeptin population on hypothalamic GnRH pulse generator activity. Additionally, we have recently shown, through neurotoxic lesioning of the MePD, that this region is implicated in puberty timing and reproductive cyclicity in female rats [24]. The present study examined the influence of maternal obesity on *Kiss1* mRNA expression in the hypothalamus and MePD of pre-pubertal and young adult rat offspring. We also determined the effects of kisspeptin antagonism in the MePD on pubertal timing, estrous cyclicity and the preovulatory LH surge in female rats exposed to a normal diet throughout their life course.

## Materials and methods

### Animals

All procedures were conducted in accordance with the United Kingdom Home Office Animals (Scientific Procedures) Act 1986. The protocols were approved by the Committee on the Ethics of Animal Experimentation of King’s College London. Adult female Sprague-Dawley rats obtained from Charles River (Margate, UK) were used as breeders in our facility at King’s College London; producing animals for study 1 and 2. A separate group of adult female Sprague-Dawley rats (Charles River) fed a normal diet throughout their life course was used in study 3. The rats were housed under controlled conditions (12 h light, 12 h dark cycle, lights on at 0700 h; temperature 22°C ± 2°C) with *ad libitum* access to food and water.

### Study 1: Effects of maternal diet-induced obesity on kisspeptin expression in the offspring

Female Sprague-Dawley rats were fed either a standard chow diet (RM3, Special Dietary Services, Essex, UK) or a highly palatable energy-dense obesogenic diet consisting of 20% animal lard, 10% simple sugars, 28% polysaccharide, and 23% protein (w/w); energy 4.5 kcal/g (Special Dietary Services) and supplemented *ad libitum* with sweetened condensed milk [∼55% simple sugars and 8% fat, 8% protein (w/w); Nestlé] and fortified with mineral and vitamin mix (AIN 93G; Special Diets Services). The animals were maintained on these diets for 6 weeks before mating, during pregnancy and lactation. The effects of these diets on maternal phenotype has been described previously [25]. Litter size was standardised to 8 pups (4 male, 4 female) 48 hours after birth. All offspring were weaned at postnatal day (pnd) 21 and subsequently fed RM1 diet *ad libitum*. In subsequent experimental groups, 2 males and 2 females from each litter were used.

#### Tissue dissection and quantitative RT-PCR

Male and female offspring of control (OffCon) and obese (OffOb) dams were culled on pnd 30 (OffCon, n = 8 per sex; OffOb, n = 10 per sex) or 100 (OffCon, n = 8 per sex; OffOb, n = 10 per sex) for determination of *Kiss1* mRNA expression in ARC, AVPV and MePD. These offspring were prepubertal and without significant difference in body weight at pnd 30 as described previously [25]. Animals were killed by decapitation and brains rapidly removed, snap frozen on dry ice and stored at -80°C until processing. Brains were cut into 300μm thick coronal sections using a cryostat (Bright Ltd., Cambridgeshire, UK) and mounted on coated polysine slides (ThermoFisher Scientific, Braunschweig, Germany). Brain punches were taken using the micropunch method [26,27] with coordinates obtained from the rat brain atlas of Paxinos and Watson [28]. For both ARC and AVPV nuclei, a single midline punch (1 mm diameter) was taken from bregma -1.7 to 3.9 and 0.26 to -1.3 respectively, while bilateral punches (0.6 mm diameter) from the MePD were taken from bregma -2.2 to -3.6. The punched sections were fixed with formalin and stained with crystal violet to confirm correct punch positioning under a microscope.

Total RNA was extracted from the punched ARC, AVPV and MePD tissues for each rat using TRI reagent (Sigma-Aldrich, Poole, UK) and reverse transcribed using the reverse transcriptase Superscript II (Invitrogen, Carlsbad, CA, USA) and random primer following the manufacturer’s instructions. Hypoxanthine phosphoribosyltransferase 1 (*Hprt1*) mRNA was used as a reference gene for normalization of target gene. The primers used for quantitative real-time PCR are shown in Table 1. The primer pairs selected were designed to amplify across at least one intron, ruling out the possibility of identical size bands resulting from genomic DNA amplification. Reaction conditions for *Kiss1* were optimized to give the best results for the primer and for the different quantities of target in samples [29]. The sample cDNA prepared as above was used as a template for the PCR using the Applied Biosystems® ViiA™ 7 Real-Time PCR System. During PCR, the amplified cDNA products were detected after each annealing phase in real time using the QuantiTect fast SYBR Green kit (QIAGEN, Hilden, Germany). Each reaction included 2 μl sample cDNA (optimized so that sample values of the PCR product were within the standard curve), 0.8 μl each of 10 μM antisense and sense primers, 4 μl QuantiTect SYBR Green mix, and 0.4 μl water to give a total reaction volume of 8 μl. The PCR cycling conditions for *Kiss1* were initial denaturation and activation at 95°C for 5 min, followed by 40 cycles of denaturation at 95°C for 10 sec and annealing at 56°C for 10 sec and 72°C for 10 sec. The *Hprt1* reaction conditions were 5 min at 95°C for one cycle, then 10 sec at 95°C, 10 sec at 55°C, and 10 sec at 72°C for 40 cycles. Expression level of *Kiss1* was determined by the threshold cycle (Ct) value in the exponential phase of the PCR reaction and normalized to the respective *Hprt1* Ct value using the comparative Ct method [30].

**Table 1:** Oligonucleotide primer pairs used for RT-PCR amplification of Kisspeptin (Kiss1) and hypoxanthine phosphoribosyltransferase 1 (Hprt1).

	Oligonucleotide primers (5’-3’)	Expected size (bp)	Reference
*Kiss1*			
Forward	TGGCACCTGTGGTGAACCCTGAAC	202	NM181692.1
Reverse	ATCAGGCGACTGCGGGTGGCACAC		
*Hprt1*			
Forward	GCAGACTTTGCTTTCCTTGG	81	NM012583
Reverse	CGAGAGGTCCTTTTCACCAG		

### Study 2: Effect of kisspeptin antagonism in the MePD on puberty timing

Post-weanling female Sprague-Dawley rats fed a normal diet prior to pregnancy and during pregnancy and lactation were used to determine the effect of MePD kisspeptin antagonism on pubertal timing. All surgical procedures were carried out under a combination of ketamine (Vetalar, 600 mg/kg, i.p.; Pfizer, Sandwich, UK) and xylazine (Rompun, 60 mg/kg, i.p.; Bayer, Newbury, UK) anesthesia. On pnd 21, animals were anaesthetized and secured in a David Kopf stereotaxic frame and small holes drilled into the skull at a location above the MePD after a small incision of the scalp. A 28-gauge cannula (Plastics One, Roanoke, VA, USA) was fitted bilaterally towards the MePD. The stereotaxic coordinates for implantation of the cannulae previously optimized [24] were 2.5 mm posterior to bregma (AP), 3.2 mm lateral (ML), and 7.8 mm below the surface of the dura (DV) using the rat brain atlas of Paxinos and Watson [28]. An osmotic mini-pump (Model 2002; Alza Corp, Mountain View, CA, USA) prefilled with kisspeptin receptor antagonist (Peptide 234; Sigma Adrich) or artificial cerebrospinal fluid (aCSF) was attached to the cannula with silicone tubing, and implanted subcutaneously (s.c.) in the interscapular space. Rats received peptide 234 (2 nmol in 6 μl/d; n = 11) or aCSF (n = 8) via the osmotic mini-pump at the rate of 0.25 μl/h for 14 days and were weighed every 3^rd^ day. Recently, there has been a debate on the blockade of kisspeptin signaling by peptide 234 [31], nonetheless, we and others have repeatedly documented the effectiveness of this peptide as a potent kisspeptin receptor antagonist in rats [23,32,33]. Rats were monitored daily for vaginal opening and first estrous (markers of puberty onset). Correct cannula placement in the MePD was confirmed by microscopic inspection of 30 μm brain sections. Only data from animals with correct cannula placement were analyzed.

### Study 3: Effects of kisspeptin antagonism in the MePD on estrous cyclicity and proestrus LH surge in adult females

Adult female rats (100 days old) obtained from Charles River were implanted with osmotic mini-pumps for bilateral intra-MePD injection of Peptide 234 (2 nmol in 6 μl/d; n = 13) or aCSF (n = 9) for a 14 day period as described above with stereotaxic coordinates being 3.14 mm posterior to bregma (AP), 3.4 mm lateral (ML) and 8.6 mm below the surface of dura (DV). Estrous cycle was monitored daily through vaginal cytology for 22 days and normal estrous cyclicity was defined as having at least 2 consecutive normal estrous cycles, which lasted for 4–5 days with 1–2 days of estrus. The cycle length was determined by the number of consecutive days from the last day of a cornified smear to the last day of an estrus smear in the subsequent cycle. Each rat was also fitted with an indwelling cardiac catheter via the jugular veins, to facilitate serial blood sampling for LH measurement. The catheters were exteriorized at the back of the head and enclosed within a 30-cm metal spring tether (Instec Laboratories, Boulder, CO, USA) secured to the slotted screw [34]. The distal end of the tether was attached to a fluid-filled swivel (Instec Laboratories), which allowed the rat to move freely around the enclosure. On the day of experimentation, rats were attached via the cardiac catheter to a computer-controlled automated blood sampling system for the intermittent withdrawal of 25 μl blood samples without disturbing the animals [35]. Blood sampling commenced at 1300 h on the day of proestrus and samples were collected every 30 min for 7 h to determine LH surges. Correct cannula placement in the MePD was confirmed by microscopic inspection of 30 μm brain sections. Only data from animals with correct cannula placement were analyzed.

### Radioimmunoassay for LH measurement

A double-antibody RIA supplied by the National Hormone and Peptide Program (Torrance, CA, USA) was used to determine LH concentrations in the 25-μl whole-blood samples. The sensitivity of the assay was 0.093 ng/ml. The interassay variation was 6.8% and the intraassay variations was 8.0%.

### Statistical analysis

Comparison between groups were made by subjecting data to one-way analysis of variance (ANOVA) and Dunnett’s test. Data are presented as the mean ± S.E.M. P < 0.05 was considered statistically significant.

## Results

### Study 1: Maternal obesity regulates kisspeptin expression in rat offspring

**Fig 1:**
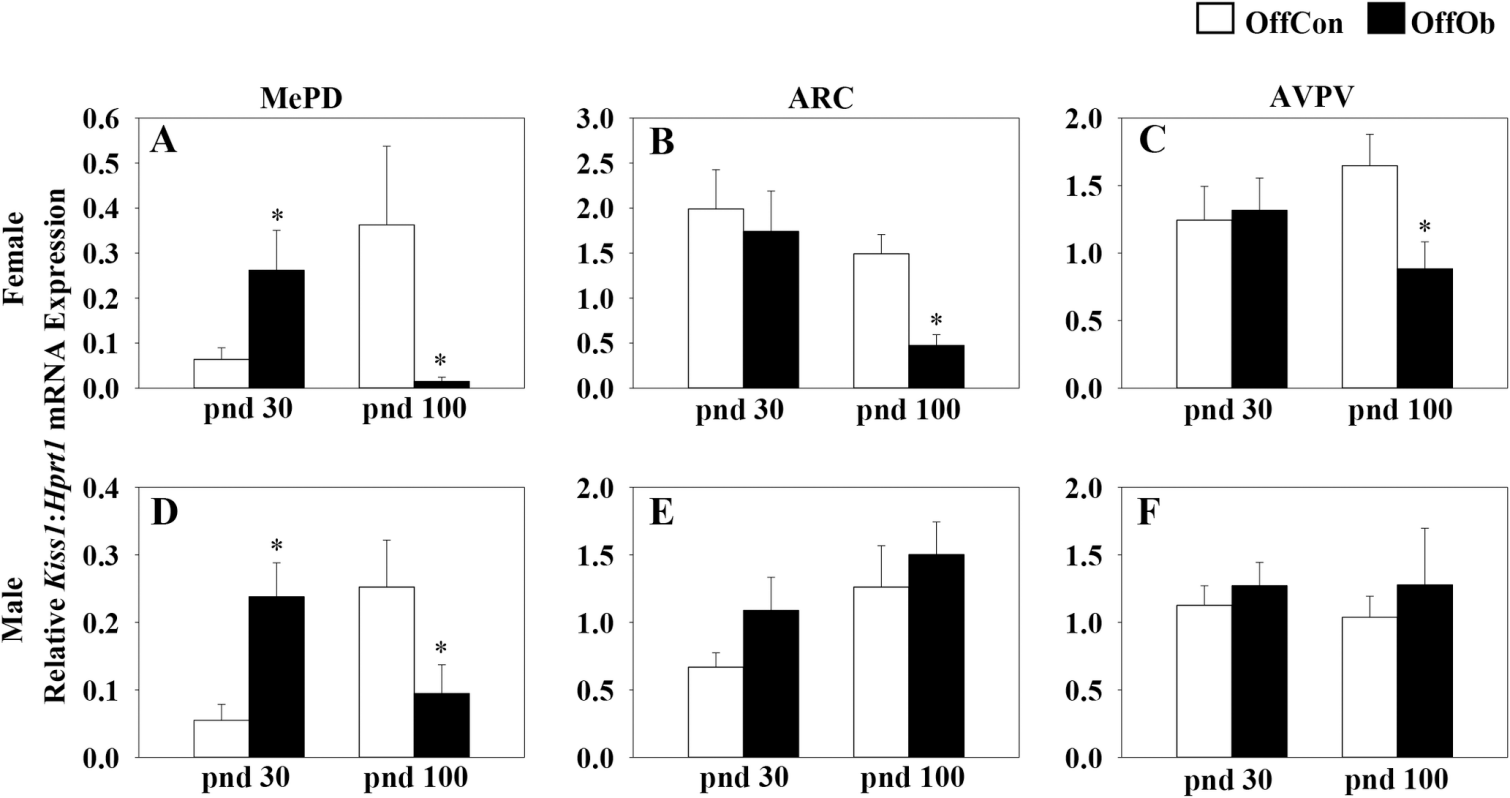
Effect of maternal obesity on *Kiss1* mRNA expression in MePD, ARC and AVPV on postnatal day (pnd) 30 and 100. Both male and female offspring of obese dams (OffOb) showed a significant higher level of *Kiss1* mRNA in the posterodorsal medial amygdala (MePD) on pnd 30 compared with offspring of control dams (OffCon) (A and D). No significant difference was observed in *Kiss1* mRNA expression in the hypothalamic arcuate (ARC) or anteroventral periventricular (AVPV) nuclei at this age (B, C, E, and F). In contrast, by pnd 100, maternal obesity reduced *Kiss1* mRNA expression in the MePD of both males and females (A and D) and similarly reduced *Kiss1* expression in the ARC and AVPV of female offspring (B and C), but not in the males (E, F). *p<0.05 *vs* OffCon, (n = 8–10 per group). Values are expressed as a ratio of *Kiss1* to *Hprt1* mRNA.

Maternal obesity increased *Kiss1* mRNA expression in the MePD of pnd 30 male and female offspring, but was without effect in the ARC or AVPV nuclei (Fig 1A–1F). In contrast, maternal obesity reduced *Kiss1* mRNA expression in the MePD of both males and females at pnd 100 (Fig 1A and 1D). Maternal obesity similarly reduced *Kiss1* expression in the ARC and AVPV of female offspring at pnd 100, but not in the males (Fig 1B, 1C, 1E and 1F).

**Fig 2:**
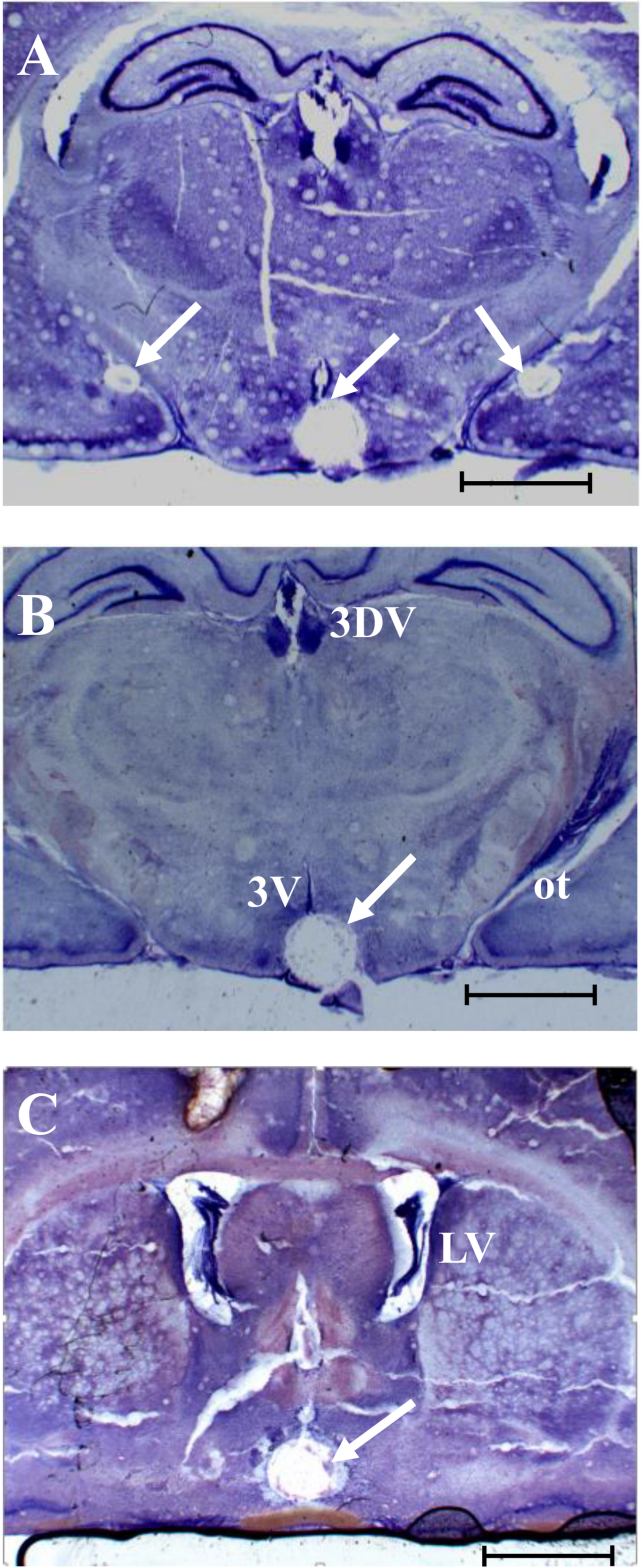
Representative photomicrograph of punched brain sections. Representative photomicrograph of 300 μm thick coronal brain sections stained with crystal violet showing the position of punched posterodorsal medial amygdala (MePD) at approximately -3.14 from bregma (A), hypothalamic arcuate nucleus (ARC) at -3.14 and -3.30 mm from bregma (A and B) and anteroventral periventricular nucleus (AVPV) taken at approximately -0.46 mm from bregma (C). Arrows point to punched brain region. Lateral ventricles (LV), optic tract (ot), third ventricle (3V), and the dorsal third ventricle (D3V) are also indicated. Scale bar; 3mm.

Representative photomicrographs showing the position of the micro-punched MePD, ARC and AVPV in the 300 μm thick coronal brain sections are shown in Fig 2.

### Study 2: Antagonism of MePD kisspeptin delays puberty onset

**Fig 3:**
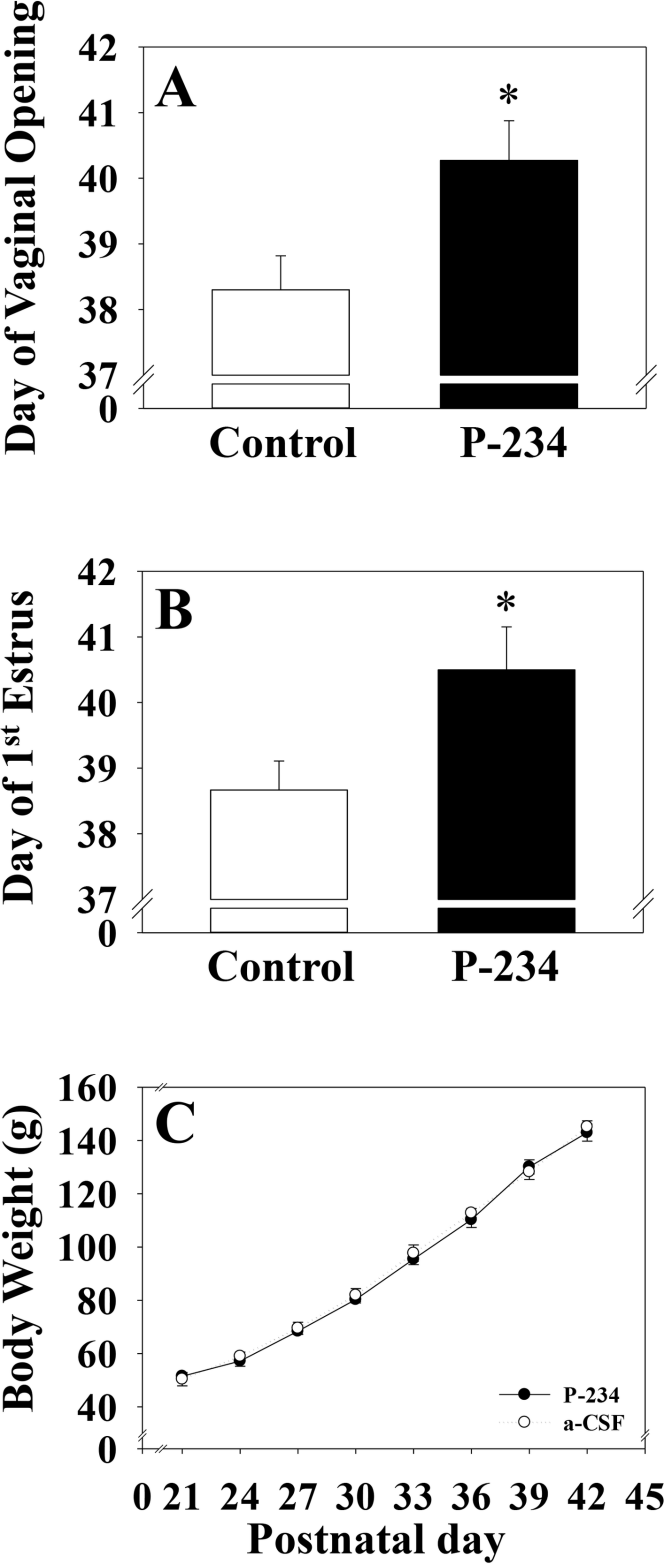
Day of vaginal opening, first estrus and body weight gain following intra-MePD infusion of Kisspeptin receptor antagonist. Bilateral micro-infusion of a kisspeptin receptor antagonist (Peptide 234; 2 nmol in 6 μl/d for 14 days, starting on postnatal day 21) into the MePD via osmotic mini-pump resulted in a significant delay in puberty onset, evidenced by day of vaginal opening (A), first estrus (B) without any significant change in body weight (C). *p<0.05 *vs* control, (n = 6–8 per group). Results represent mean ± S.E.M.

Micro-infusion of the kisspeptin receptor antagonist (Peptide 234) into the MePD of post-weanling female rats significantly delayed day of vaginal opening (aCSF; 38.3 ± 0.52 vs P-234; 40.3 ± 0.60) and first estrus (aCSF; 38.7 ± 0.44 vs P-234; 40.5 ± 0.65), both external markers of puberty onset (Fig 3A and 3B). Body weight gain was comparable between both groups (Fig 3C).

**Fig 4:**
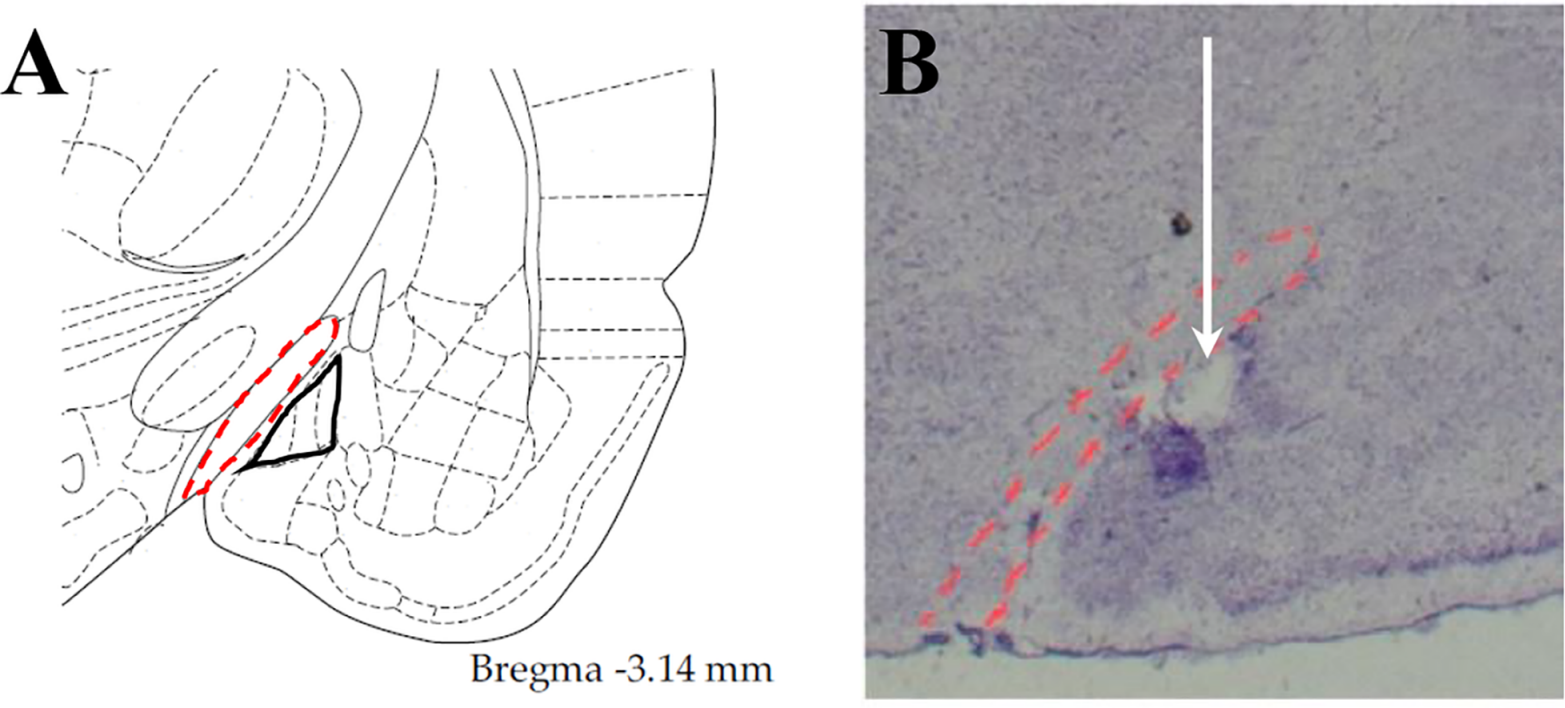
Confirmation of the cannula position within the MePD. Diagram from the rat brain atlas of Paxions and Watson [28] showed the location of the MePD (marked) in relation to the optic tract (dot marked) (A). Cresyl violet stained brain slice in a representative animal showed accurate cannula position within the MePD. Arrow indicates the site corresponding to the tip of cannula (B).

Representative photomicrograph of a 30 μm coronal brain section showing correct cannula placement in the MePD is presented in Fig 4.

### Study 3: MePD Kisspeptin antagonism disrupts estrous cycle and reduces the incidence of LH surges in adult females

**Fig 5:**
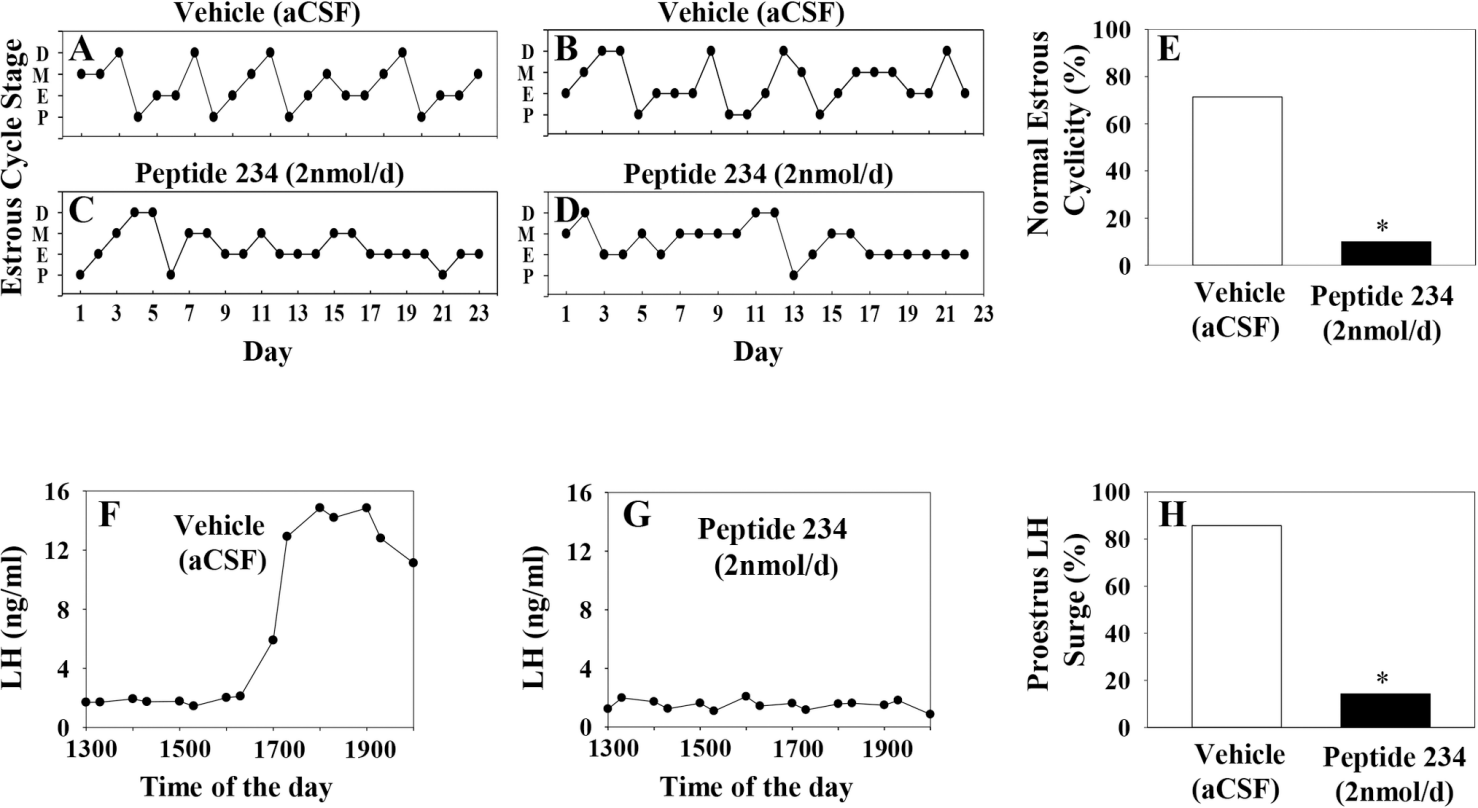
Effect of chronic intra-MePD administration of kisspeptin receptor antagonist on estrous cyclicity and proestrous LH surge. Representative examples of estrous cycle pattern (A—D) and LH profile (F and G) in rats treated with kisspeptin receptor antagonist (Peptide 234; 2 nmol in 6 μl/d for 14 days, starting on day 1), or vehicle (aCSF, artificial cerebrospinal fluid). Kisspeptin antagonism in the MePD significantly reduced the percentage of rats showing normal estrous cycles (E) as well as incidence of proestrus LH surges (H). *p<0.05 *vs* control, (n = 7–10 per group).

Antagonism of kisspeptin receptors in the MePD significantly disrupted estrous cyclicity resulting in a reduced percentage of rats showing normal estrous cyclicity; 10% of antagonist treated rats showing normal estrous cycle patterns compared with 71% of controls (Fig 5A–5E). In addition, the incidence of proestrus LH surges in kisspeptin receptor antagonist-treated rats was significantly reduced compared with vehicle. Only 1 out of 10 animals treated with antagonist exhibited LH surge at proestrus, while 6 out of 7 showed LH surges in vehicle treated animals (Fig 5F–5H).

## Discussion

Kisspeptin is essential for puberty initiation and coordination of reproductive function in adulthood. In the present study, maternal obesity exerted a profound influence on MePD *Kiss1* expression in both pre-pubertal and young adult offspring. The functional significance of which is inferred by antagonism of MePD kisspeptin signaling resulting in delayed puberty, disruption of estrous cyclicity and reduced occurrence of preovulatory LH surges in normal female offspring. The pattern of postnatal *Kiss1* expression as a result of exposure to maternal obesity, first an increase in expression in prepubertal life, followed by a decrease in adulthood, parallels the reproductive phenotype of offspring of obese mothers, who first display puberty advancement followed by disruption in reproductive function and behavior in adulthood [8,11].

A causal relationship exists between maternal obesity and early onset of puberty in rats [5,8]. Although the kisspeptinergic system of the offspring has been shown to be modified by maternal undernutrition [36], there are as yet, no established report with respect to maternal over-nutrition. In the present study, maternal obesity robustly upregulated MePD *Kiss1* mRNA expression in pre-pubertal male and female offspring, whereas *Kiss1* expression was not affected in the ARC and AVPV at this age; an unexpected finding given the notable role of hypothalamic kisspeptin in the control of puberty, given the established link between a pre-pubertal increase in hypothalamic kisspeptin signaling and occurrence of puberty [37,38]. A recent study has shown that maternal obesity raises endogenous estradiol level in postnatal day 30 offspring [16], opening the possibility of *Kiss1* regulation by sex-steroid. However, it remain a conundrum why in the present study AVPV *Kiss1* wasn’t upregulated by maternal obesity whereas MePD *Kiss1* was, given that both kisspeptin population are regulated by estradiol in a similar manner [39,40]. Perhaps differential neuroendocrine modulation of these brain areas by maternal obesity may be a factor to consider. A similar experimental paradigm involving over-nutrition by litter size reduction also did not affect ARC and AVPV *Kiss1* expression [41]. In contrast, other reports showed increased *Kiss1* expression in ARC in response to postnatal over-nutrition [42,43]. These inconsistencies may derive from differences in study design and dietary regimen. Notwithstanding these disparities in experimental outcome, the possibility of a time-dependent increase in hypothalamic *Kiss1* expression [44] cannot be ruled out, which may not be captured at postnatal day 30 when *Kiss1* mRNA was quantified in the current study.

The role of MePD as a neurobiological locus for puberty timing is not unprecedented; albeit having previously been suggested to have an inhibitory influence. This is driven by the fact that most neuronal projections from the MePD to the reproductive related hypothalamic nuclei express gamma–aminobutyric acid (GABA) [45], which is inhibitory to puberty [46]. In addition, neurotoxic lesioning of the MePD advances puberty onset in female rats [24]. Micro-infusion of GABA into the medial hypothalamic preoptic area (mPOA) or ARC has also been shown to suppress pulsatile LH release [37,38]. However, the important interaction between GABA and kisspeptin should be recognised; GABAergic neurotransmission in the mPOA of female rats can be modulated by kisspeptin [47] and kisspeptin’s activation of its G protein-coupled receptors attenuate the effect of GABA by desensitizing GABA_B_ receptors on GnRH neurons [48]. Kurian et al. [49] have also noted that kisspeptin signaling partly mediates the reduction in the GABAergic tone that precedes the reactivation of GnRH neurons during puberty onset in monkeys [48]. Therefore, the upregulation of MePD *Kiss1* expression as a result of exposure maternal obesity not only reflects the anatomical changes that occur in the medial amygdala during puberty transition [50], but may functionally imply that kisspeptin mediates reduction in GABAergic tone crucial for puberty onset.

Understanding the functional significance of the increased MePD *Kiss1* expression by maternal obesity may be furthered by our study of antagonism of MePD kisspeptin signaling in female rats fed a normal diet throughout their life course. Bilateral injection of a kisspeptin antagonist into the MePD delayed puberty onset, evidenced by delay in vaginal opening and first estrus, which was independent of changes in body weight. These data not only corroborate earlier report on delayed puberty onset in female rats infused intracerebroventricularly with kisspeptin antagonist [33], but further suggest that MePD *Kiss1* expression may modulate GnRH release and provide evidence for the contribution of MePD kisspeptin signaling in pubertal advancement. Although the MePD lies upstream of the hypothalamus, it remains to be determined if the delay in puberty onset by MePD kisspeptin antagonism implies a hierarchical role for MePD kisspeptin neurons over their hypothalamic counterparts. This may be a likely scenario, since the MePD sends out projections innervating the mPOA, AVPV and ARC [45,51] but there are no reciprocal projections from either ARC or AVPV to the MePD [52]. Further to the synaptic connection between MePD kisspeptin neurons and cell bodies of GnRH neurons in the mPOA [20], we have shown that MePD kisspeptin signaling modulates GnRH pulse generator frequency in female rats [23]. It has been proposed that the reawakening of the GnRH pulse generator at puberty may involve either extra-hypothalamic signals regulating this neural oscillator or a direct intrinsic hypothalamic mechanism [20]. The former supports a higher-order function for the MePD on puberty timing, given the tonic inhibitory brake exerted by amygdaloid GABAergic projections to hypothalamic reproductive nuclei [51], the loss of which facilitates puberty onset [24]. The present study not only suggests a key role for MePD kisspeptin in the circuitry controlling puberty onset, but opens up future directions for the regulation of GnRH pulse generator by extra-hypothalamic signals.

Since the MePD is a critical site for lesion-induced obesity in rats [53] as well as puberty timing [24], it may equally be sensitive to metabolic cues for pubertal timing. Our earlier studies have shown that maternal obesity elicits an exaggerated and prolonged neonatal leptin surge compared to control offspring [25] which is thought to be a developmental cue in normal hypothalamic development [54]. A key site for leptin’s regulation of reproduction is the hypothalamic ventral premammilary nucleus (PMv) [55], which is largely populated by excitatory glutamatergic neurons [56] and expresses virtually no GABAergic component [57]. Within the entire mouse hypothalamus, only PMv fibres project significantly to the MePD [52]. Leptin-induced excitatory output from the PMv may therefore be responsible for the increased MePD *Kiss1* expression in the pre-pubertal offspring as observed in the current study.

In adult offspring, maternal obesity resulted in considerable reduction in *Kiss1* mRNA levels in all three brain regions examined in the female offspring, but only in the MePD of male offspring. The reduced MePD *Kiss1* expression in the male offspring may provide mechanistic explanation for reduced LH levels and altered sexual behaviour reported in offspring of obese dams [10,11], supported by recent reports on the key role of kisspeptin in the limbic brain on sexual and emotional behaviour in male rats [58] and men [59]. Maternal high fat nutrition during pregnancy and lactation has also been associated with irregularity in estrous cycles in rat offspring [8]; a common phenomenon being persistent estrus. The reduction in hypothalamic kisspeptin signaling is accompanied by a decline in ovulatory capacity [47], while a moderate knockdown of kisspeptin signaling in the AVPV increased the time spent in estrus and metestrus stages of the estrous cycle as well as reduction in the incidence of spontaneous LH surge in rats [60]. The present results suggest that the disruption in reproductive functions by maternal obesity [7,8] may be causally linked to reduced *Kiss1* expression in both hypothalamic nuclei and the MePD, which was particularly profound in adult female offspring. It therefore appears that maternal obesity shortens the reproductive life-cycle of the offspring by targeting the reproductive brain, specifically by decreasing kisspeptin availability which is essential for regulating LH release and ovulation [61,62]. Whether this is translatable to humans remain to be proven, although there is substantial supportive evidence linking early menarche to early menopause in women [63] and maternal obesity driving the former in girls [3].

In view of the known role of hypothalamic kisspeptin signaling in estrous cyclicity and LH surges [60], we ventured to investigate whether MePD kisspeptin is also a key component in the preovulatory LH surge generation. Interestingly, bilateral intra-MePD injection of kisspeptin receptor antagonist reduced the percentage of animals showing normal estrous cycles and spontaneous LH surges in a comparable fashion to kisspeptin knockdown in the AVPV [60]. The loss of normal estrous cyclicity after MePD kisspeptin antagonism is in keeping with the prolonged estrous cycles found after MePD lesioning in rats [24]. These data, in addition to the high expression of estrogen receptor in the MePD [21] further suggest the involvement of the MePD in the cascade relaying estrogenic feedback mechanism important for estrous cyclicity and the LH surge. Additionally, vasopressin fibers of suprachiasmatic nucleus (SCN) origin convey circadian information important for preovulatory LH surges to AVPV kisspeptin neurons [64]. Similarly, vasopressin fibers form close apposition with MePD kisspeptin neurons [20], but it is not known whether these vasopressin fibers also originate from the SCN, whereby kisspeptin antagonism may have desynchronized the neural circuitry controlling the LH surge.

In conclusion, these data provide evidence of a key role for the MePD in pubertal timing and reproductive function and suggests that maternal obesity may act via MePD kisspeptin signaling to influence reproductive function in the offspring.
